# A peptide containing the receptor binding site of insulin-like growth factor binding protein-2 enhances bone mass in ovariectomized rats

**DOI:** 10.1038/s41413-018-0024-9

**Published:** 2018-08-14

**Authors:** Gang Xi, Christine Wai, Clifford J. Rosen, David R. Clemmons

**Affiliations:** 1 Department of Medicine, University of North Carolina at Chapel Hill, Chapel Hill, USA; 2 Maine Medical Center Research Institute, Scarborough, USA

## Abstract

Male *Igfbp2*−*/*− mice have a significant reduction in bone mass and administration of a peptide that contains the insulin-like growth factor binding protein-2(IGFBP-2) receptor-binding domain stimulates bone formation in these animals. Female *Igfbp2*−/− mice do not have this phenotype but following ovariectomy (OVX) lose more bone than OVX wild-type mice. This suggests that in the absence of estrogen, IGFBP-2 is required to maintain bone mass. Therefore these studies were undertaken to determine if this peptide could stimulate bone acquisition in OVX rats. OVX rats were divided into seven treatment groups: sham animals, OVX animals, OVX animals receiving a control scrambled peptide, or one of three doses of the active peptide termed PEG-HBD-1 (0.7, 2, and 6 mg·kg^-1^) and an OVX group receiving parathyroid hormone (PTH) (50 µg·kg^-1^ per day). The peptides were administered for 8 weeks. DXA revealed a significant reduction in femoral and tibial areal bone mineral density (aBMD) after OVX, whereas treatment with the high-dose peptide increased aBMD by 6.2% ± 2.4% (*P* < 0.01) compared to control peptide; similar to the increase noted with PTH (5.6% ± 3.0%, *P* < 0.01). Similar increases were noted with two lower doses of the peptide (3.8% ± 1.5%, *P* < 0.05 for low dose; 3.1% ± 1.6%, *P* = 0.07 for middle dose). Micro CT showed that the OVX control peptide animals had reductions of 41% and 64% in femoral trabecular BV/TV and trabecular number, respectively. All three doses of the peptide increased bone volume/total volume (BV/TV) significantly, while the low and middle doses increased trabecular number. Cortical BV/TV and thickness at the midshaft increased significantly with each dose of peptide (18.9% ± 9.8%, *P* < 0.01 and 14.2% ± 7.9%, *P* < 0.01 for low dose; 23.7% ± 10.7%, *P* < 0.001 and 15.8% ± 6.1%, *P* < 0.001 for middle dose; 19.0% ± 6.9%, *P* < 0.01 and 16.2% ± 9.7%, *P* < 0.001 for high dose) and with PTH (25.8% ± 9.2%, *P* < 0.001 and 19.4% ± 8.8%, *P* < 0.001). Histomorphometry showed that the lowest dose of peptide stimulated BV/TV, trabecular thickness, mineral apposition rate (MAR), bone formation rate/bone surface (BFR/BS), number of osteoblasts/bone perimeter (N.ob/B.pm), and decreased osteoclast surface/bone perimeter (Oc.S/B.Pm). The highest dose stimulated each of these parameters except MAR and BFR/BS. Thus, the heparin-binding domain receptor region of IGFBP-2 accounts for its anabolic activity in bone. Importantly, this peptide enhances bone mass in estrogen-deficient animals.

## Drug discovery: peptide boost for bone mass

An experimental peptide stimulates bone acquisition in female rats who have had their ovaries removed, raising the prospect a new drug for osteoporosis.

IGFBP-2 is an insulin-like growth factor (IGF) binding protein, which regulates the amount of IGF-I and II that are transported out of the blood and are available to influence the growth and proliferation of bone-producing osteoblasts. Previous studies have suggested that IGFBP-2 is required to maintain bone mass in the absence of estrogen, and that a 13 amino acid peptide (HBD1) from the core of the protein could provide a substitute for it.

In this study, David Clemmons at the University of North Carolina at Chapel Hill and his colleagues demonstrate that injecting the peptide into ovariectomized female rats prompts significant increases in bone mass, whereas control animals lost bone.

## Introduction

Insulin-like growth factor binding protein-2(IGFBP-2) is a high affinity form of an insulin-like growth factor (IGF)-binding protein and has the capacity to regulate the amount of IGF-I and IGF-II that are transported out of the vasculature, and thus able to interact with cell surface receptors.^1^ Therefore, it can function through that mechanism to modulate IGF actions.^2^ The addition of molar excess IGFBP-2 inhibits the ability of IGF-I to stimulate osteoblast proliferation in short-term tissue culture experiments.^3^ However, the addition of IGF and IGFBP-2 together in equimolar concentrations stimulates osteoblast growth.^4^ In addition to controlling IGF access to receptors, IGFBP-2 binds directly to a cell surface receptor termed receptor tyrosine phosphatase beta (RPTPβ).^5^ IGFBP-2 binding to RPTPβ stimulates osteoblast differentiation and bone formation.^6^ The IGFBP-2/ RPTPβ signaling pathway functions coordinately with the IGF-I receptor-linked signaling pathway. Following IGFBP-2 binding to RPTPβ the AKT inhibitor phosphatase and tensin homolog (PTEN) is inactivated, thereby accentuating the ability of IGF-I to stimulate AKT activation, an important driver of osteoblast differentiation.^7^ The region of IGFBP-2 that binds to RPTPβ has been determined and is contained in the central core of the protein sequence.^5^ A synthetic peptide containing this core 13 amino acid sequence activates the RPTPβ linked pathway and promotes bone acquisition in vivo.^6,8^ The importance of these findings was demonstrated in male *Igfbp2* −/− mice that had reduced areal bone mass, decreased trabecular bone volume/total volume (BV/TV), and low bone turnover.^9^ Administration of the 13 AA peptide enhanced bone formation and inhibited bone resorption in *Igfbp2*−/− mice.^8^ However, in contrast to males, female mice did not exhibit this phenotype. To further investigate this difference, we prepared female *Igfbp2* −/− mice with and without ovariectomy (OVX) and compared the degree of bone loss to wild-type C57BL6J control and ovariectomized mice.^10^ Although OVX in wild-type mice caused a significant reduction in BV/TV, trabecular number and increased trabecular spacing, the deletion of IGFBP-2 in ovariectomized mice caused a significantly greater decrease in trabecular BV/TV and reduced trabecular number in the proximal femur. Those mice also demonstrated a significantly lower mineral apposition rate as well as bone formation rate, and the interaction between OVX and loss of IGFBP-2 was significant. Those results suggested that in the absence of estrogen, IGFBP-2 was required to maintain bone mineral density. Since the 13 amino acid peptide can substitute for intact IGFBP-2, and in the absence of estrogen, IGFBP-2 appears to be necessary for maintenance of skeletal health, we undertook this study to determine if administration of this peptide to ovariectomized rats would prevent estrogen-deficiency-induced bone loss.

## Results

**Fig. 1:**
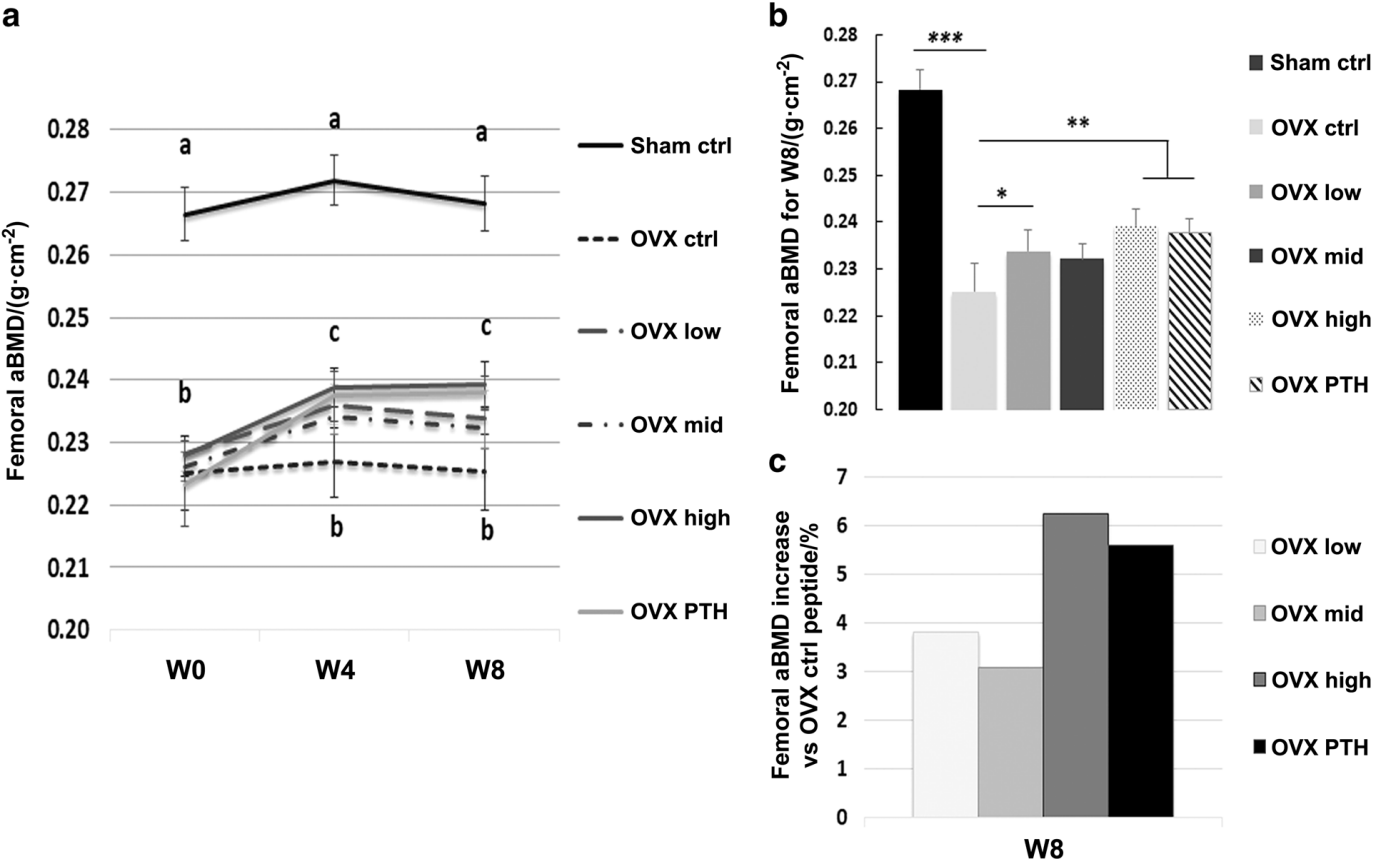
PEG-HBD1 peptide stimulates femoral aBMD in OVX rats. **a** Femoral aBMD values were obtained from different groups of rats using a DXA scanner at the beginning (W0), 4 weeks (W4), and 8 weeks (W8) of peptide treatment. Each data point represents mean ± SEM for each group animals at different time points. A significant difference was detected between sham ctrl rats and OVX ctrl rats at all three time points (indicated by the letter a). No difference was detected in all OVX rat treatment groups at baseline (W0) or after control peptide treatment at W4 and W8 time points (indicated by the letter b). Significant differences were detected between OVX rats treated with all three dose of PEG-HBD1 or PTH and OVX rats treated with a control peptide at W4 and W8 time points (indicated by the letter c, at least *P* < 0.05) except the mid dose of PEG-HBD1 at W8 time point. **b** Each bar graph represents mean ± SEM of each group of animals after 8 weeks of treatment. ***P < 0.001, **P < 0.01, and *P < 0.05 indicated the significant difference between two treatments. **c** Each bar represents the percentage increase in femoral aBMD for that treatment compared to control (ctrl) peptide-treated OVX rats

The rats which had been ovariectomized 8 weeks prior to beginning the peptide injections consistently weighed more than the sham control rats. All groups of rats gained weight during the study; and the ovariectomized animals maintained a higher weight compared to the sham controls (Supplemental Fig. 2). Analysis of areal bone mineral density (aBMD) in the femur showed that the sham animals had significantly greater aBMD compared to OVX animals at the initiation of the injection period, and this difference remained unchanged. Sham animals that received the control peptide or high-dose PEG-HBD1 (data not shown) had no significant change in aBMD. All OVX groups significantly had lower aBMD (*P* < 0.001) than the sham controls at baseline (for example 15.4% reduction) (Fig. 1a). Following 8 weeks of treatment with PEG-HBD1 peptide, the OVX high-dose peptide treatment group showed a significant increase (*P* < 0.05) in femur aBMD compared to either their baseline value or the OVX control peptide-treated animals (Fig. 1b). This change was apparent following 4 weeks of treatment and persisted for the 8 week treatment interval. Both low-and mid-dose PEG-HBD1 peptide treatment groups at 4 weeks, but only low-dose peptide treatment at 8 weeks, showed significant increases in the femoral aBMD compared to the control peptide-treated rats. When the data were expressed as percent increase over basal, the greatest response (e.g. 6.2%) increase was noted in the high-dose peptide-treated animals, with the next greatest response 5.6% in the parathyroid hormone (PTH) treated group. Following intermittent exposure this PTH preparation stimulated osteoblast differentiation, which was comparable to a PTH preparation obtained for a different source (Supplemental Fig. 3). The response in the OVX low-dose group (3.8% ± 1.5% increase, *P* < 0.05) was significantly greater than control peptide (Fig. 1c). The mid-dose group increased 3.1% ± 1.6% but this change was not significant. Analysis of tibial aBMD showed no significant increases at any dose. All three doses of PEG-HBD1 peptide stimulated an increase in both femoral and tibial bone mineral content (BMC) compared to the control peptide-treated groups. The percentage increase in femoral BMC varied from 3.6% to 7.2% (Table 1), and tibial BMC varied from 3.5% to 9.8% (Table 2). Sham control, sham high dose (data not shown), and OVX control peptide-treated animals showed no change. PTH increased aBMD at the femur (5.6% ± 2.1%, *P* < 0.01) but not the tibia (1.4% ± 3.2%, *p*, NS) when compared to OVX control peptide responses. Analysis of the femoral BMC after 8 weeks showed significant increases in each of the treatment groups (e.g., 0.407 ± 0.042, *P* < 0.05, for low dose; 0.404 ± 0.038, *P* < 0.01 for middle dose; 0.418 ± 0.044, *P* < 0.01 for high dose; 0.418 ± 0.025, *P* < 0.01 for PTH vs. 0.390 ± 0.010 for control peptide) (Table 1). The high-dose treatment group response was significantly greater than the lower-dose and middle-dose groups, and it was comparable to PTH. Analysis of tibial BMC showed that the low-dose and high-dose groups had significantly greater responses, whereas the middle-dose group did not achieve significance. The response in the PTH group was comparable to the low-dose and high-dose responses (Table 2).

**Table 1:** Bone mineral content of femur

Items	0 Week/g	4 Weeks/g	8 Weeks/g
Sham ctrl	0.457 ± 0.030	0.465 ± 0.031	0.470 ± 0.034
OVX ctrl	0.400 ± 0.030^a^	0.395 ± 0.049^a^	0.390 ± 0.001^a^
OVX low	0.396 ± 0.046	0.409 ± 0.049^b^	0.407 ± 0.042^b^
OVX middle	0.398 ± 0.033	0.394 ± 0.031	0.404 ± 0.038^c^
OVX high	0.403 ± 0.039	0.414 ± 0.049^c^	0.418 ± 0.044^c^
OVX PTH	0.391 ± 0.021	0.412 ± 0.025^b^	0.418 ± 0.025^c^

^a^*P* < 0.01 compared to sham control (ctrl)

^b,c^*P* < 0.05, *P* < 0.01 compared to OVX control (ctrl)

**Table 2:** Bone mineral content of tibia

Items	0 Week/g	4 Weeks/g	8 Weeks/g
Sham ctrl	0.417 ± 0.034	0.424 ± 0.025	0.425 ± 0.034
OVX ctrl	0.387 ± 0.045^a^	0.380 ± 0.048^b^	0.375 ± 0.051^b^
OVX low	0.385 ± 0.031	0.406 ± 0.041^c^	0.412 ± 0.044^e^
OVX middle	0.382 ± 0.026	0.391 ± 0.027	0.388 ± 0.025
OVX high	0.390 ± 0.034	0.411 ± 0.032^d^	0.409 ± 0.023^d^
OVX PTH	0.390 ± 0.026	0.408 ± 0.024^d^	0.409 ± 0.018^d^

^a,b^*P* < 0.01, *P* < 0.05 compared to sham control (ctrl)

^c,d,e^*P* < 0.05_,_ *P* < 0.01, *P* < 0.001 compared to OVX control (ctrl)

**Fig. 2:**
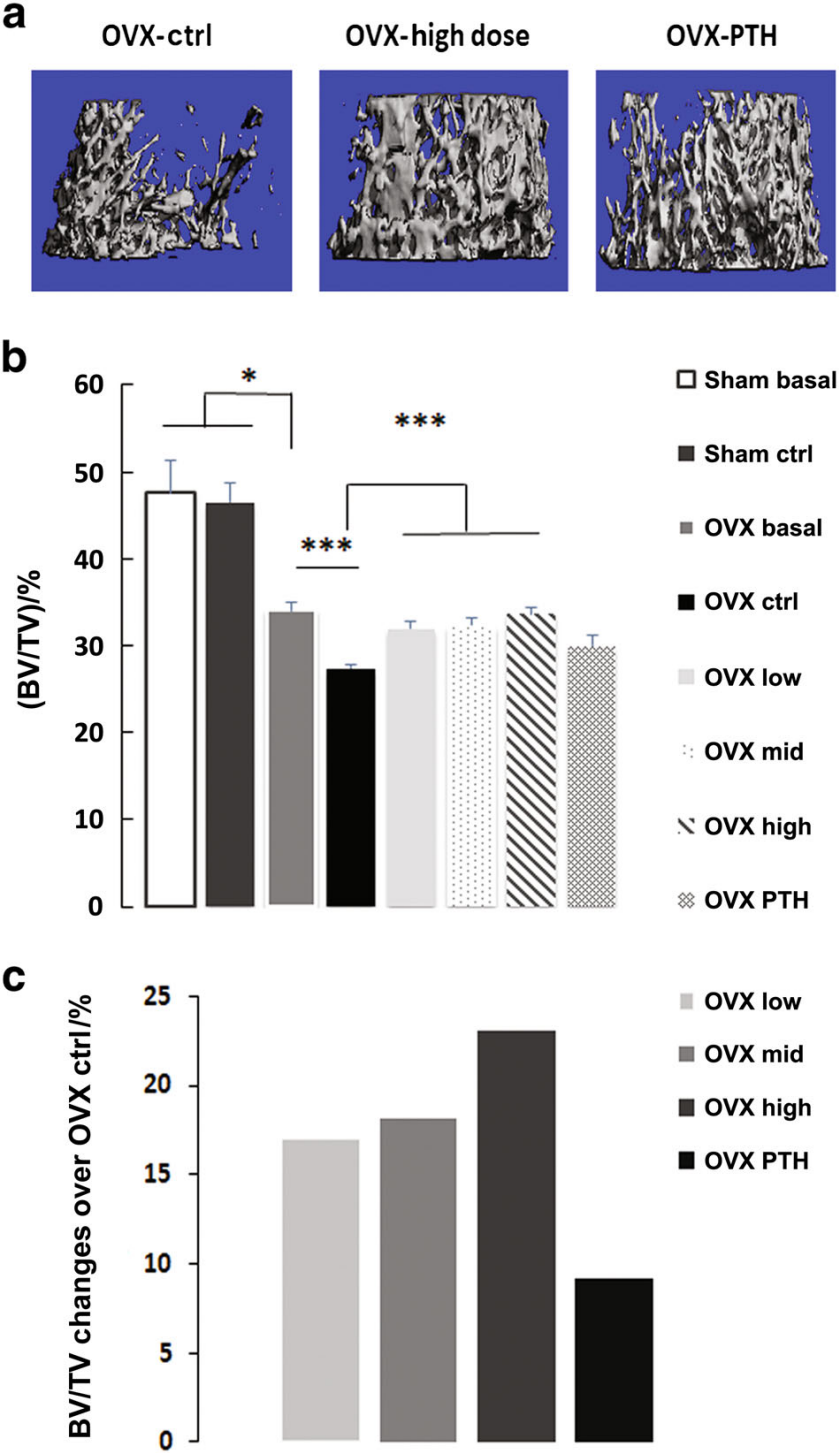
PEG-HBD1 peptide stimulates BV/TV of trabecular bone in the femur of OVX rats. **a** Representative images of femoral trabecular bone OVX rats treated with control (ctrl), high dose PEG-HBD1 peptide or PTH. **b** Each bar represents the mean ± SD of BV/TV of trabecular bone in femur from different group of animals. ***P < 0.001 and *P < 0.05 indicated the significant difference between two treatments. **c** Each bar represent the mean of BV/TV percentage change over control peptide (ctrl) treated rats by a different dose of peptide or PTH

Micro CT data obtained after 8 weeks of treatment showed that the changes in femoral trabecular bone BV/TV results were consistent with the aBMD results. Representative photographs are shown in Fig. 2a. The analysis showed that low dose of the PEG-HBD1 peptide resulted in a statistically significantly greater BV/TV (e.g. 32% ± 2%) compared to control peptide-treated OVX animals (e.g., 27% ± 1%) (Fig. 2b). Similarly, the middle dose of peptide resulted in a greater value (e.g. 32% ± 2%), and the greatest value was noted with the highest dose (e.g. 34% ± 2%). These values were significantly greater than the OVX control peptide-treated animals. PTH increased BV/TV but the change was not significant. Of note the OVX control peptide-treated animals lost significant amounts of bone (e.g., 28.3% reduction, *P* < 0.001) during the study compared to their baseline. All of the sham surgery groups were significantly greater than the OVX animals (sham basal vs. OVX basal or sham treated with control peptide vs. OVX treated with control peptide) (Fig. 2b). When the data were expressed as percent increase over OVX control, the greatest response (e.g. 22.7% ± 7.3% increase, *P* < 0.001) was noted in the high-dose peptide-treated animals, while two lower doses were also effective (e.g. 16.7% ± 6.6% increase for low dose, *P* < 0.001; 18.2% ± 5.8% for middle dose, *P* < 0.001) (Fig. 2c). Changes in connectivity were of a similar magnitude in the low and mid-dose groups, but were only significant in the low-dose group (e.g., 9.94% ± 4.03 vs. 7.68% ± 3.68, *P* < 0.05). PTH also induced a significant increase in connectivity (Table 3). Analysis of trabecular number also showed significant increases in the low dose PEG-HBD1 treated group (e.g. 12.6%) as well as the mid-dose treated group (17.9%). The high-dose treated group and PTH animals did not show significant increases (Table 3). Trabecular thickness did not increase significantly in any treatment group (Table 3). As for the BV/TV, the OVX animals followed over time had a significant reduction in connectivity density and trabecular number compared to start of the study; and the sham treated groups were significantly greater than the OVX controls for these parameters (Table 3). All doses of peptide but not PTH increased the total bone volume density significantly in OVX rats (Table 3). Analysis of cortical changes showed that all three doses of the PEG-HBD1 peptide stimulated significant increases is BV/TV (18.9% ± 9.8%, *P* < 0.01 for low dose; 23.7% ± 10.7%, *P* < 0.001 for middle dose; 19.0% ± 6.9%, *P* < 0.01 for the high dose). PTH induced a 25.2% ± 9.2% increase (*P* < 0.001) (Table 4). Significant increases in cortical thickness were also detected in all peptide-treated groups (14.2% ± 7.9%, *P* < 0.01 for low dose; 15.8% ± 6.1%, *P* < 0.001 for middle dose; 16.2% ± 9.7%, *P* < 0.001 for the high dose), and PTH stimulated a 19.6% ± 8.8% increase (*P* < 0.001) (Table 4).

**Table 3:** Comparison of different treatments on trabecular bone in Sham and OVX rats

Items	Sham basal	Sham ctrl	OVX basal	OVX ctrl	OVX low	OVX mid	OVX high	OVX PTH
Conn.D/mm^-^^3^	69.94 ± 28.23	40.47 ± 8.77^a^	10.71 ± 3.76^a^	7.68 ± 3.68	9.94 ± 4.03^f^	9.70 ± 5.19	8.66 ± 3.05	9.55 ± 4.36^g^
Trabecular number/mm	4.94 ± 0.31	4.08 ± 0.33^a^	2.52 ± 0.24^c^	1.46 ± 0.23^d^	1.64 ± 0.20^g^	1.72 ± 0.47^f^	1.50 ± 0.27	1.54 ± 0.25
Trabecular thickness/mm	0.19 ± 0.07	0.29 ± 0.02^a^	0.39 ± 0.03^a^	0.40 ± 0.02	0.39 ± 0.05	0.42 ± 0.05	0.44 ± 0.06	0.39 ± 0.03
Total volume density/(mg·mL-1)	424 ± 58	420 ± 48	327 ± 39^b^	260 ± 19^e^	317 ± 29^h^	322 ± 23^h^	336 ± 38^h^	283 ± 42

^a,b,c^*P* < 0.01, *P* < 0.05, *P* < 0.001 compared to sham basal

^d,e^*P* < 0.01, *P* < 0.001 compared to OVX basal

^f,g,h^*P* < 0.05, *P* < 0.01, *P* < 0.001 compared to OVX control (Ctrl)

**Table 4:** Comparison of different treatments in cortical bone

Items	(BV/TV)/%	Cortical thickness/mm
OVX ctrl	53.5 ± 7.1	0.592 ± 0.044
OVX low	63.6 ± 5.0^a^	0.676 ± 0.061^a^
OVX mid	66.2 ± 3.4^b^	0.686 ± 0.043^b^
OVX high	63.7 ± 2.9^a^	0.688 ± 0.047^b^
OVX PTH	67.0 ± 2.5^b^	0.708 ± 0.022^b^

^a,b^*P* < 0.01, *P* < 0.001 compared to OVX control (ctrl)

**Fig. 3:**
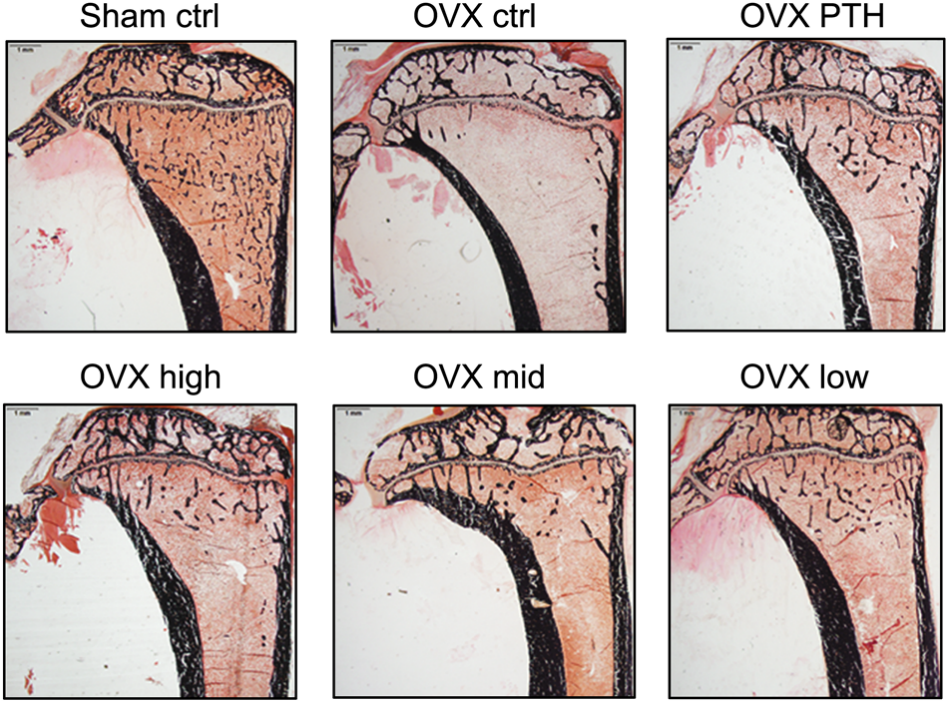
Von Kossa staining of undecalcified sections from tibia of OVX rats. Representative images of Von Kossa stained sections from different groups of rats. Four-um-thick sections were stained

**Fig. 4:**
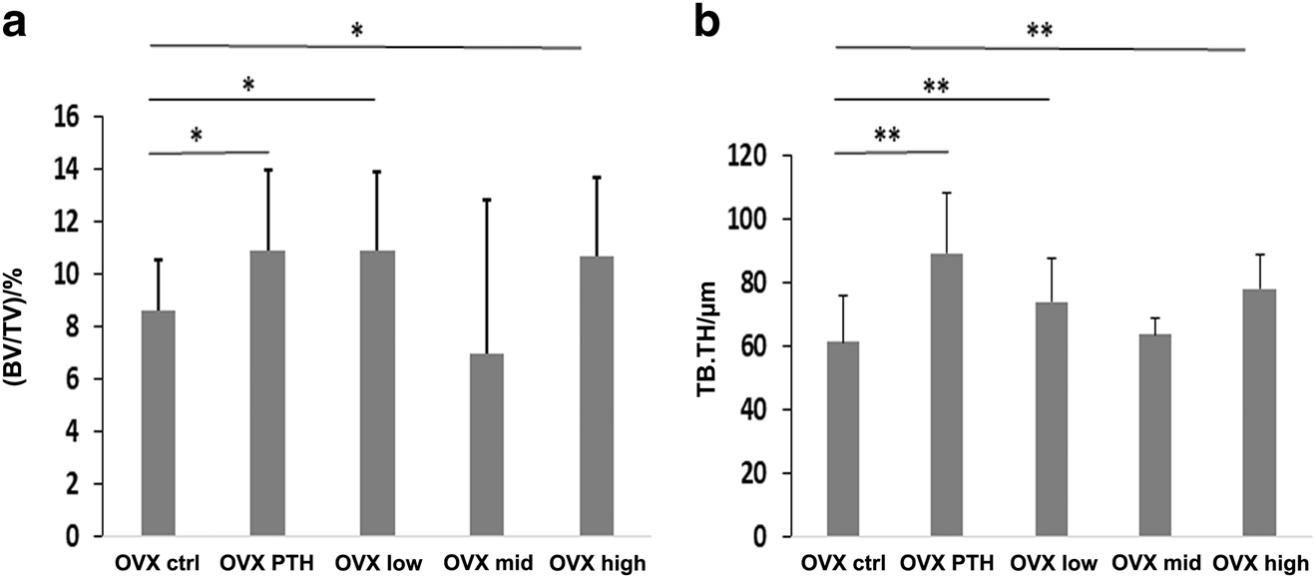
Histomorphometric analysis of the effect of the PEG-HBD1 peptide on bone volume/total volume (BV/TV) and trabecular thickness (TB.TH) of tibia in OVX rats. **a**, **b** Each bar graph represents the mean ± SD of BV/TV **(a)** or trabecular bone thickness (**b**) of tibia from the OVX rats with different treatments. **P < 0.01 and *P < 0.05 indicates a significant difference between two treatments

**Fig. 5:**
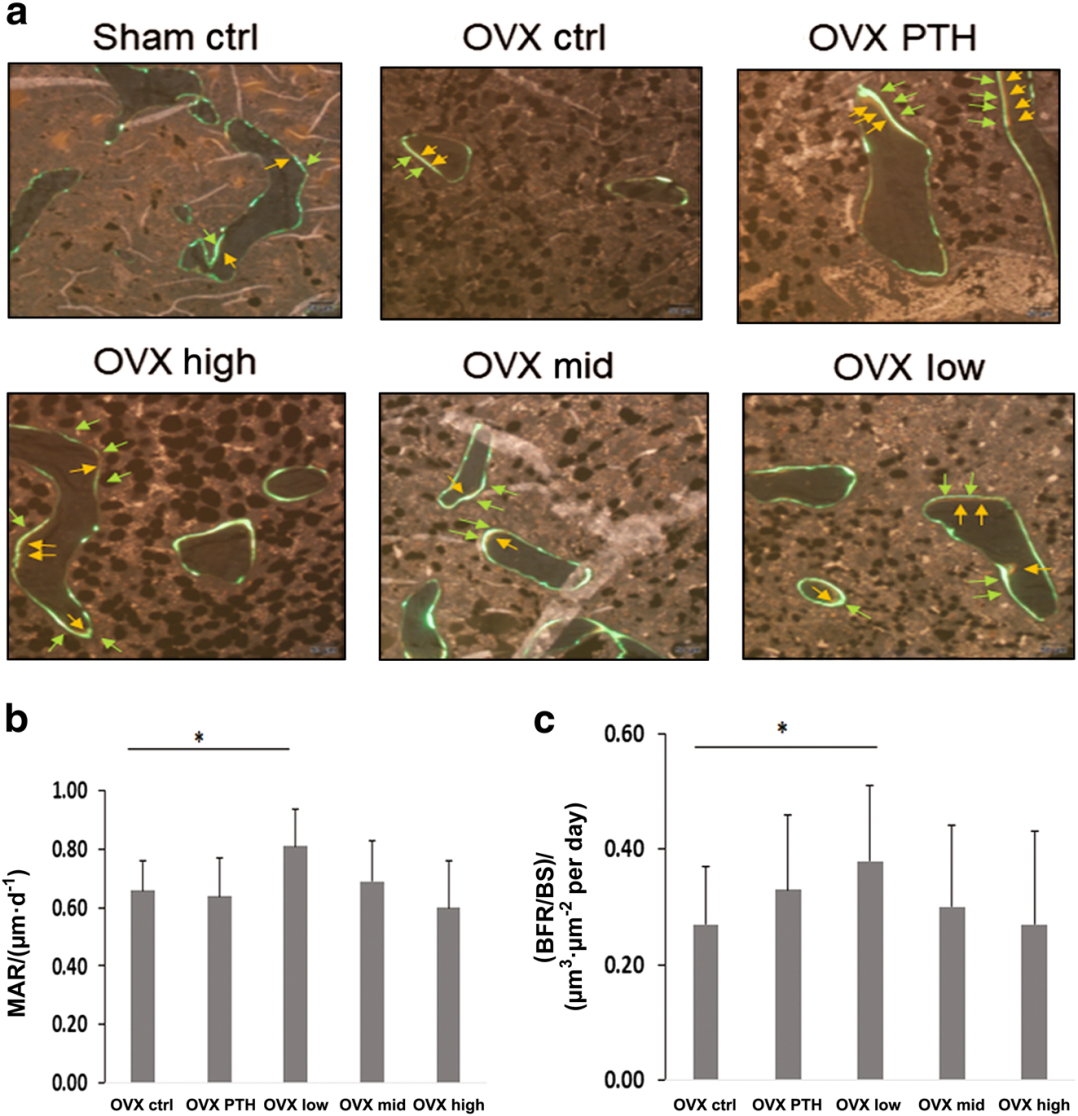
Histomorphometric analysis of the PEG-HBD1 peptide on mineral apposition rate (MAR) and bone formation rate (BFR) in tibia. **a** Representative images of demeclocycline and calcein staining, which showed the structural and dynamic change in the tibia from OVX rats after different treatments. **b, c** Each bar graph represents the mean ± SD of MAR (**b**) or BFR/bone surface (BS) (**c**) in the tibia from OVX rats with different treatments. *P < 0.05 indicates a significant difference between two treatments

**Fig. 6:**
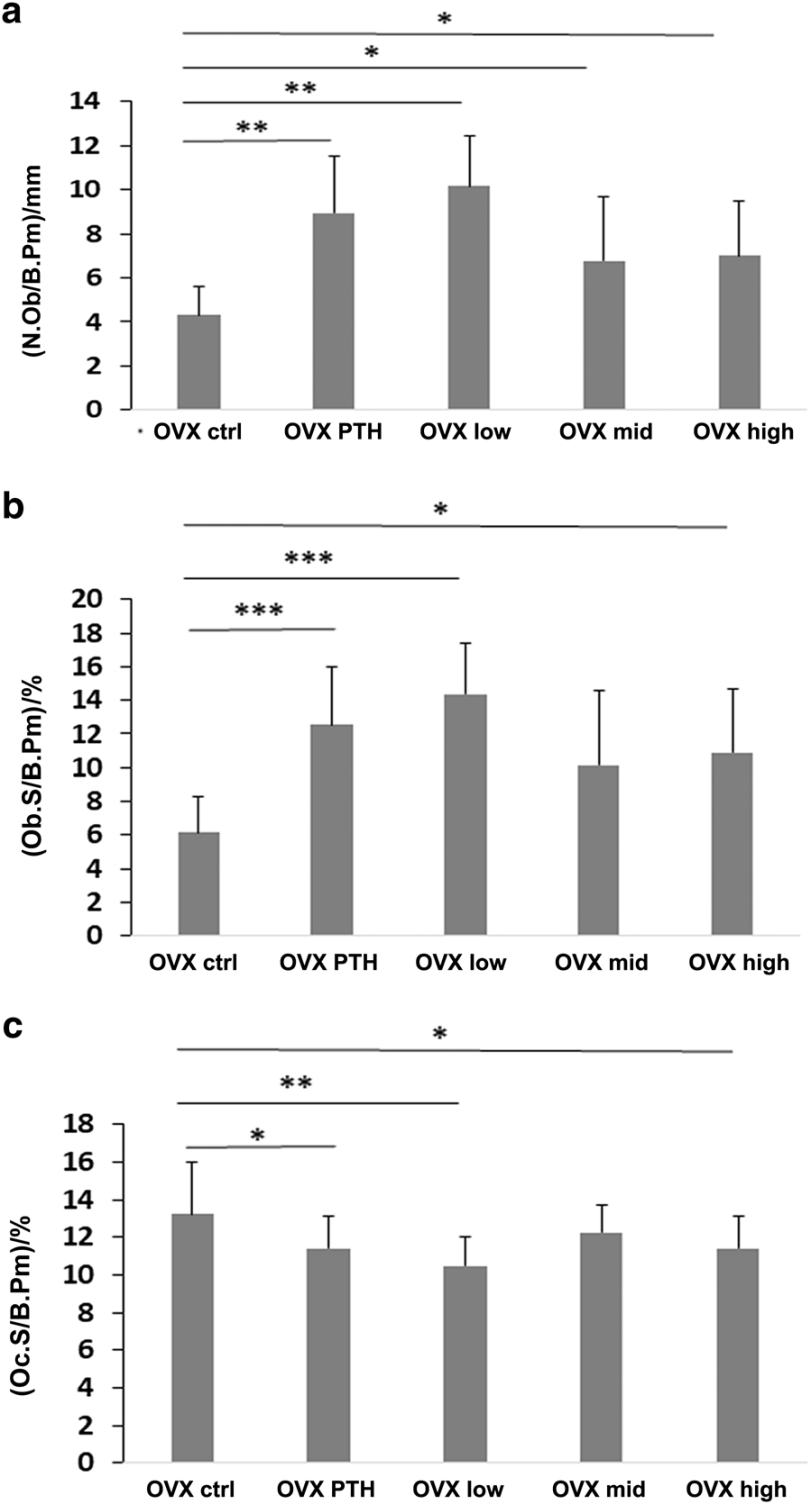
PEG-HBD1 peptide increased osteoblasts number (N.Ob) and surface (Ob.S) but decreased osteoclasts surface (Oc.S) in tibia from OVX rats. Each bar graph represents the mean ± SD of N.Ob per bone perimeter (B.Pm) (**a**) or Ob.S/B.Pm (**b**) or Oc.S/B.Pm (**c**) in the tibia from OVX rats with different treatments. ***P < 0.001, **P < 0.01, and *P < 0.05 indicated the significant difference between two treatments

Histomorphometry showed that both PTH and the PEG-HBD1 peptide stimulated bone formation (Fig. 3). As can be seen from the figure the mineralization front was widened following these treatments and Von Kossa staining showed increased trabecular formation. Quantitative analysis showed that PTH, low and high dose of the PEG-HBD1 peptide stimulated BV/TV significantly compared to control (10.89% ± 3.06% for PTH, *P* < 0.05; 10.91% ± 2.97% for low dose, *P* < 0.05; 10.67% ± 3.05% for high dose, *P* < 0.05 vs. 8.60% ± 1.98% for control peptide) (Fig. 4a). Similarly PTH and the low and high doses of peptide also stimulated trabecular thickness (89.28 μm ± 19.02 µm for PTH, *P* < 0.01; 73.92 μm ± 13.71 µm for low dose, *P* < 0.01; 77.95 μm ± 11.04 µm for high dose, *P* < 0.01 vs. 61.19 μm ± 14.57 µm for control peptide) (Fig. 4b). Analysis of mineral apposition rate showed that it was stimulated by the low-dose of PEG-HBD1 peptide, but PTH and the mid-and high doses had no effect (Fig. 5a, b). The low-dose PEG-HBD1 peptide and PTH stimulated bone formation rate/bone surface (BFR/BS) to the same extent, but the mid-dose and high-dose groups did not change significantly (Fig. 5c). Analysis of the number of osteoblasts or osteoblast surface per bone perimeter area showed that the low-dose peptide and PTH had the greatest effect, but high dose of the peptide also stimulated both parameters significantly, whereas the mid-dose of the peptide only stimulated the former (Fig. 6b). Analysis of osteoclast surface per unit bone perimeter showed that PTH as well as low and high-dose peptide induced significant decreases (Fig. 6c).

**Fig. 7:**
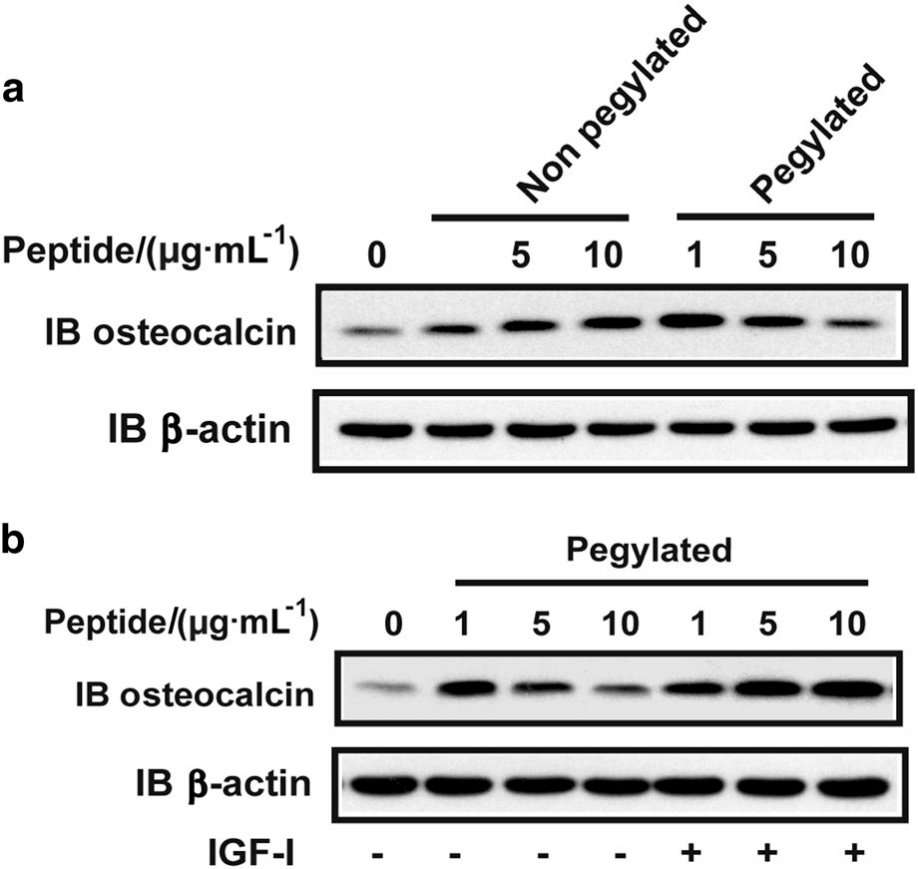
PEG- but not non PEG-HBD1 peptide had reverse dose response in stimulating osteocalcin expression in osteoblasts. **a**, **b** Cells were cultured as described in the materials and methods. Cell lysates were harvested and immunoblotted with an anti-osteocalcin antibody. The blots were reprobed with an anti-β-actin antibody as a loading control. The concentration of IGF-I was 100 ng·mL^-1^

To further probe why the low dose was more potent than the mid-and high doses, we analyzed osteoblast differentiation in vitro. Comparison of the pegylated and non-pegylated forms of the HBD1 peptide showed that when added to cultures in concentrations between 1–10 µg·mL^-1^ the pegylated peptide was associated with a reversing dose response curve in stimulating osteoblast differentiation as assessed by osteocalcin secretion. In contrast, the non-pegylated form of the peptide did not result in reversing dose response curve (Fig. 7a). Importantly, this reversing dose response induced by the pegylated peptide disappeared when adequate IGF-I (100 ng·mL^-1^) was present (Fig. 7b).

## Discussion

Our initial studies conducted in *Igfbp2* −/− mice showed that only male mice developed a low bone mass phenotype.^9^ Those animals had reduced BV/TV, decreased bone formation rate, and markedly reduced mineralization. In subsequent studies, we demonstrated that the 13 amino acid peptide (HBD1) that contained a region from the central core of IGFBP-2 could substitute for the intact protein in stimulating osteoblast proliferation and enhancing bone formation in *Igfbp2* −/− mice.^8^ We further showed that this peptide bound to a specific receptor on the osteoblast surface, RPTPβ, a membrane-associated receptor that contains tyrosine phosphatase activity in its cytoplasmic domain.^5^ Following HBD1 peptide binding to RPTPβ a signal transduction pathway is induced that results in the enhancement of IGF-I stimulated AKT activation.^6^ This occurs because RPTPβ directly dephosphorylates PTEN.^5^ Ligand occupancy of RPTPβ induces polymerization, which inactivates the phosphatase, thereby resulting in enhanced PTEN tyrosine phosphorylation.^11^ This inactivates PTEN, thus enhancing the degree of AKT activation that occurs in response to IGF-I. Since PTEN and the AKT pathway have been shown to play an important role in bone acquisition,^12^ we subsequently determined that the peptide could stimulate both the differentiation of MC-3T3 cells as well as primary calvarial osteoblasts.^13^ We reported that this effect is mediated by IGFBP-2 or the HBD1 peptide binding to RPTPβ. Further support for the activity of this peptide was derived from mutagenesis studies in which intact IGFBP-2 that had residues in the HBD1 sequence altered to reduce binding to RPTPβ did not stimulate osteoblast differentiation.^6^

Because of its ability to stimulate bone formation in mice, we were encouraged to test this peptide in wild-type rats. In contrast to males, female *Igfbp2−/−* mice had a normal bone phenotype.^9^ These findings suggested that estrogen had an interaction with IGFBP-2, or that estrogen was inducing a factor that could substitute for IGFBP-2. A subsequent study demonstrated that deletion of IGFBP-2 in ovariectomized mice resulted in a further reduction in BV/TV and bone formation rate compared to ovariectomized wild-type control mice.^10^ Based on those observations, we chose to determine the efficacy of the HBD1 peptide on osteoblast function in ovariectomized rats.

That OVX was successful in altering the bone phenotype was proven by dual-energy x-ray absorptiometry (DXA) and micro-computed tomography (micro CT) analysis, which showed marked reductions in aBMD, and BV/TV as well as trabecular number compared to sham controls. Similarly, histomorphometry showed significant reduction in both these parameters as well as mineral apposition rate and a large increase in osteoclast number per bone perimeter surface. Furthermore over the 2-month study interval following OVX, there was a further reduction in BV/TV that was significant, as well as reductions in connectivity density and trabecular number, indicating an ongoing loss of bone due to estrogen deficiency. Treatment with the synthetic peptide was highly successful in reversing several of these changes. Specifically analysis of aBMD showed that all three doses of peptide caused significant increases in the femur in both BMD and BMC. Tibial BMC was also significantly increased, although the deficit that occurred as a result of OVX was not completely corrected with any treatment. Micro CT analysis further confirmed and extended the data. All three doses of synthetic peptide caused a significant increase in BV/TV, although only the low dose was effective in reversing the changes in connectivity density. Similarly, the low and mid doses of the peptide were very effective in increasing trabecular number as compared to OVX animals treated with control peptide. Micro CT studies also showed that all three doses of peptide stimulated significant increases in cortical thickness. Since this parameter was also reduced by OVX, the results support the conclusion that the peptide is capable of reversing this important change, which is a good predictor of breaking strength.

Histomorphometry further confirmed these findings, however, only the low dose of peptide was effective on most of these parameters. Specifically that dose of peptide induced significant increases in BV/TV, mineral apposition rate, bone formation rate, number of osteoblasts per bone perimeter, and a significant reduction in osteoclast surface per bone perimeter. The high dose of peptide increased trabecular thickness, the number of osteoblasts per surface parameter, and osteoblast surface per bone perimeter percent, but it did not induce significant increases in the other parameters. Therefore, the low dose of the peptide appeared to be the most effective stimulant of bone formation. PTH significantly increased BV/TV, trabecular thickness, and number of osteoblast per perimeter surface. These findings suggest that the PEG-HBD1 peptide is effective in stimulating bone acquisition in non-genetically manipulated, ovariectomized rats.

The mechanism by which IGFBP-2 induces partial recovery from the effect of OVX is not entirely defined. Following overiectomy in mice Demambro et al. noted a 27% reduction in serum IGFBP-2, suggesting that part of the effect may be replacement of this deficiency.^10^ Similarly, other investigators have also shown that in rats OVX leads to a reduction in serum IGFBP-2 and in IGFBP-2 expression in calvarial osteoblasts.^14,15^ Therefore, both local and systemic IGFBP-2 may be reduced following estrogen withdrawal. However since female IGF *Igfbp2* *−*/*−* mice show no skeletal phenotype other mechanisms must be operative. A potential mechanism is suggested by the observation that IGFBP-2 utilizes RPTPβ to stimulate osteoblast differentiation. RPTPβ is known to have other ligands and one ligand, pleiotrophin, has been shown to be estrogen dependent.^16^ Furthermore, pleiotrophin transgenic mice have minimal reduction in aBMD or BV/TV in response to OVX whereas; in male transgenic mice, there was no increase in BMC, BMD, or bone formation rate.^17^ This suggests a possible mechanism to account for why the *Igfbp2 −/−* female mice do not develop a significant bone phenotype. Following OVX pleiotrophin would be significantly reduced, therefore it would be predicted that IGFBP-2 deletion would result in further bone loss in ovariectomized animals as was noted previously.^10^ Based on th`e results in this paper, we conclude that administration of the PEG-HBD1 peptide can partially correct this deficit. Sexually dimorphic changes have been noted in other components of the IGF-I axis;^18,19^ but whether there are as yet undiscovered factors such as pleiotrophin that could account for these changes has not been determined.

The explanation for loss of an effect of the mid and high doses of peptide on some of the histomorphometric parameters is not readily apparent. It should be noted these doses were not without any effect in that they did increase trabecular number and the number of osteoblasts per surface area. Investigators have noted a reversing dose response curve when assessing the response to IGFBP-2 for stimulating osteoblast differentiation. Palermo et al. demonstrated that when IGFBP-2 was added without IGF to osteoblast cultures it induced alkaline phosphatase at 1.0 nM but when added at 10 nmol·L^-1^ this effect attenuated.^20^ In contrast when they added a substantial amount of IGF-II with IGFBP-2 there was no reversing dose response. Other investigators also noted when IGFBP-2 was added without IGF-I to differentiating pre-chondrocyte cultures there was a reversing dose response curve.^21,22^ Furthermore, those investigators also utilized a C-terminal peptide from IGFBP-2 that contained the HBD1 sequence and a reduced form of intact IGFBP-2 and obtained similar results.^22^ This indicates that the effect is not simply due to binding IGF and preventing its access to receptors since neither of those peptides retained IGF-binding activity.^22^ The HBD1 peptide would behave similarly because it has no IGF-I-binding activity. Therefore based on these published findings and our data it is possible that under certain circumstances high doses of the pegylated peptide may not stimulate some parameters of bone formation unless adequate IGF-I or II is present in the microenvironment. We conclude that either those histomorphometric parameters are more sensitive to this inhibitory effect of high concentrations or that the timing of injections was such that high serum concentrations were present at time during which the labels were injected, whereas the changes in aBMD and micro CT reflect changes that occurred over the entire 2-month treatment interval. IGFBP-2 secretion by osteoblasts varies widely during differentiation,^23^ and its effects have been noted to be stage specific.^24^ Therefore, a sensitive measure such as histomorphometry may reflect more acute fluctuations in extracellular concentrations of HBD-1 and IGF-I. In contrast bone volume changes may not have been as sensitive to acute fluctuations in concentrations of these peptides, but rather require sustained intervals of stimulation of bone formation. Future studies will be required to discern the actual mechanism by which this is occurring.

Our study has significant limitations. The animals that were utilized were relatively young and therefore may have undergone some compensatory changes in response to OVX, which have been reported previously such as, increased bone formation rate, and an increase in the number of osteoblasts per bone perimeter surface.^25,26^ Studies in older animals will be required to determine if the peptide is as effective in a model in which these changes do not occur. Additionally our animals did not respond as well to PTH when compared to some studies,^27–29^ although the PTH that we used in this study had the similar biological activity when compared to PTH obtained from a different source (Supplemental Fig. 3). This difference could be due to dosing, frequency of injection (exposure time), animal dietary formulation, or animal strain differences. Previous studies have shown that PTH exerts opposite effects on osteoblast differentiation due to different exposure times^30^ or different stages of osteoblast  differentiation,^31^ therefore, it has been considered as a double-edged sword for bone metabolism.^32^ Finally, we have no functional parameters such as breaking strength to prove that these changes in morphometry and histomorphometry would translate into resistance to fracture. In spite of these limitations, we believe that the data demonstrate that this peptide shows promise as a therapeutic candidate for improving bone anabolism. Furthermore, since it reduced osteoclast number it may have the advantage of being able to inhibit resorption at the same time that it stimulates formation. Therefore, we believe further studies in other animal models and with more precisely defined dosing regimens are warranted.

These findings emphasize the point that IGF-I and IGFBP-2 and I function coordinately.^5,6^ In the absence of IGF-I excess IGFBP-2 has no anabolic effect and its ability to inhibit PTEN is IGF-I dependent. Our studies have shown that RPTPβ polymerization, which is required to inhibit PTEN activation, requires not only IGFBP-2 binding to RPTPβ but also RPTPβ association with vimentin. Vimentin association is dependent upon vimentin serine phosphorylation, which is mediated through recruitment of PKC zeta, a biochemical event that is IGF-I receptor dependent.^6^ Therefore, although this does not fully explain the reversing dose response it would explain the inability of osteoblasts to respond to high concentrations of IGFBP-2 or HBD1 peptide in the absence of adequate IGF-I. Since several physiologic variables cause major changes in IGF-I, this suggests that states of low IGF-I and high IGFBP-2 (such as malnutrition or advanced age) would have a low aBMD;^33–35^ whereas high IGF-I and high IGFBP-2 would be anabolic.^36–38^ Therefore, optimal use of this peptide may require selection of clinical conditions wherein there is maintenance of normal IGF secretion or pharmacologic manipulation to increase IGF production.

## Materials and methods

### Generation of synthetic peptides and peptides pegylation

The synthetic peptide containing the heparin-binding domain 1 (HBD1) of human IGFBP-2 (CKHHLGLEEPKKLR) and  a scrambled HBD1 peptide (CKPLRLSKEEHPLK) (control peptide) were synthesized by Genscript (Piscataway, NJ). Purity and sequence identity were confirmed by mass spectrometry. HBD1 and HBD1 control peptides (that each contained the N-terminal cysteine) were pegylated with methoxy PEG maleimide (20 000 kDa) (JenKem Biotechnology, Allen, TX) following a procedure described previously.^39^

### Animals and treatments

The animal study protocol was reviewed and approved by the Institutional Animal Care and Use Committee of University of North Carolina at Chapel Hill. Sham (*n* = 26) and OVX (*n* = 56) rats (Sprague Dawley) at age of 16 weeks were purchased from Charles River Labs (Wilmington, MA). Animals were housed at UNC facility for another 8 weeks before any treatment. At that time six of sham or OVX rats were sacrificed to obtain basal data. The rest of sham rats were assigned to one of two treatment groups: (1) PEG-HBD1 peptide (*n* = 10, 6 mg·kg^-1^ per 96 h); (2) Peg Control peptide (*n* = 10, 6 mg·kg^-1^ per 96 h). OVX rats were assigned to one of five treatment groups: (1) PEG-Control peptide (*n* = 10, 6 mg·kg^-1^ per 96 h); (2) PEG-HBD1 high dose (*n* = 10, 6 mg·kg^-1^ per 96 h); (3) PEG-HBD1 middle dose (*n* = 10, 2 mg·kg^-1^ per 96 h); (4) PEG-HBD1 low dose (*n* = 10, 0.7 mg·kg^-1^ per 96 h); (5) PTH (*n* = 10, 50 µg·kg^-1^ per 24 h). Rat PTH (1-34aa) was purchased from BACHEM (Torrance, CA). The treatments were continued for 8 weeks. The animals were weighed  weekly. To determine the frequency of peptide injection, a pilot study was undertaken in which blood samples were obtained at multiple time points after peptide injection and the serum peptide concentrations were determined. The results showed that the trough level of PEG-HBD1 peptide was reached 96 h  after the first injection and was maintained at that level after multiple injections (Supplemental Fig. 1). To measure serum peg peptide, an anti-HBD1 antibody was raised in our laboratory in a rabbit using a synthetic peptide conjugated with a KLH (Thermo Fisher Scientific, Rockford, IL) as an immunogen.

### Dual-energy x-ray absorptiometry (DXA) and micro-computed tomography (micro CT) scanning

At the initiation and following 4 and 8 weeks of injection of peptide injection, the left side of tibia and femur of animals were scanned with a DXA system (Lunar PIXImus, GE Lunar Corp.) at UNC small animal imaging center. In addition, six of sham rats and six of OVX rats were sacrificed at the initiation of the study to obtain basal micro CT data. The femurs and tibiae of all animals were harvested after 8 weeks of treatment for micro CT scanning at UNC small animal imaging center using a microCT 40 scanner (Scanco USA, Inc., Wayne, PA).

### Histomorphometric study

Double labeling was used for the histomorphometric studies. Demeclocycline (50 mg·kg^-1^) and calcein (20 mg·kg^-1^) (MillporeSigma, St. Louis, MO) were injected (i.*p*.) at 9 days and 3 days before the animals were sacrificed, respectively. The left tibias were sent to Dr. Roland Baron’s lab at Harvard School of Dental Medicine for histomorphometric  analsis. Briefly, the fixed rat tibiae were dehydrated with graded acetone and ethanol then embedded in methyl methacrylate. Undecalcified 4-um-thick sections were obtained by microtome (RM2255, Leica Biosystems, Germany) and stained with Von Kossa method for detecting mineralized bone. A consecutive second section was left unstained for the analysis of fluorescence labeling, and the third section was stained with 2% Toluidine Blue (pH 3.7) for the analysis of osteoblasts and osteoclasts. The bone histomorphometric analysis was performed in the proximal tibia under 200× magnification in a 1.8 mm high x 1.3 mm wide region 400 μm away from the growth plate using OsteoMeasure analyzing software (Osteometrics Inc., Decatur, GA, USA).

### In vitro study

MC-3T3 E1 clone 4 (CL4) cells were obtained from ATCC (Manassas, VA). Cells were cultured in α-MEM (glucose 1 000 mg·L^-1^) containing 10% fetal bovine serum (Atlanta Biological, Flowery Branch, GA). After confluency, culture medium was changed to differentiation medium (DM), which contained 10% fetal bovine serum plus 50 µg·mL^-1^ ascorbic acid and 4 mmol·L^-1^ β-glycerol phosphate. Fresh DM was applied every 72 h. Non-pegylated or PEG-HBD1 (1 or 5 or 10 µg·mL^-1^) was added when fresh DM was applied. Additional cultures were exposed to IGF-I (100 ng·mL^-1^) plus the PEG-HBD1 peptide every 72 h. For PTH treatment, cells were exposed to rat PTH (rPTH, Bachem, 50 ng·mL^-1^) or bovine PTH (bPTH, Millpore Sigma, 50 ng·mL^-1^) when fresh DM was applied. After 6 h exposure, PTH was removed by applying the fresh DM again.

The cell monolayers were lysed in a modified radioimmunoprecipitation assay buffer. Immunoblotting was performed using a dilution 1:150 for anti-osteocalcin (Santa Cruz Biotechnology, Inc. Santa Cruz, CA) and a dilution 1:5 000 for anti- β-actin (Millpore Sigma, St. Louis, MO) antibody. The proteins were visualized using enhanced chemiluminescence (Thermo Fisher Scientific, Rockford, IL).

### Statistical analysis

The results are expressed as the mean ± standard deviation (SD) or standard error of mean (SEM) as indicated. The results were analyzed for statistically significant differences using one way ANOVA followed by Tukey’s post hoc multiple comparison test. Statistical significance was set at *P* < 0.05.

## Electronic supplementary material

Supplemental materials
