# Supplementary material for: A peptide containing the receptor binding site of insulin-like growth factor binding protein-2 enhances bone mass in ovariectomized rats
Source: Bone Res. 2018 Aug 14;6:23. doi: 10.1038/s41413-018-0024-9 (PMC6089876; doi:10.1038/s41413-018-0024-9)
Supplement: Supplementary file 1 — Supplemental materials [file 41413_2018_24_MOESM1_ESM.docx]

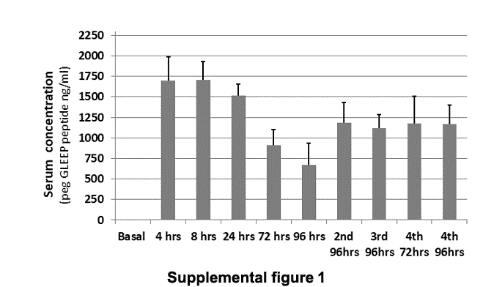


**Supplemental figure 1. Serum PEG-HBD1 peptide concentrations at different time points after injection.** Each bar graph represents the mean ± SD of serum peptide concentration at different time points after injection. The peptide concentration was measured using an anti-HBD1 antibody.

**Supplemental figure 2. Sham and OVX rats body weight change during the experiment.** Each bar graph represents the mean ± SD of body weight for each group of animals. The rats were weighed weekly.


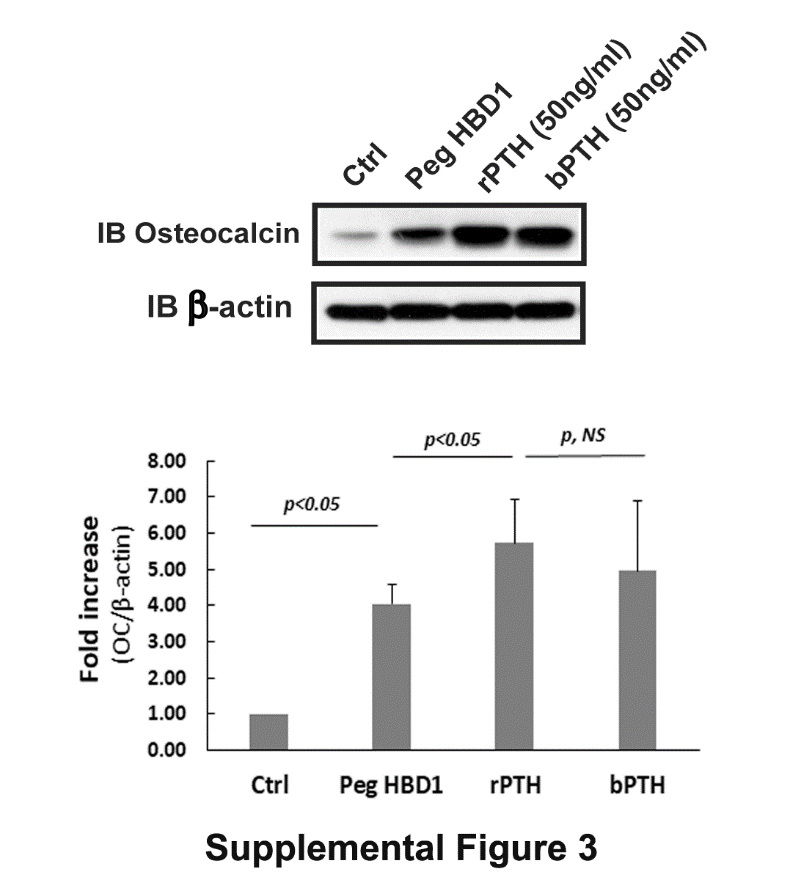


**Supplemental Figure 3: Intermittent treatment of PTH stimulates osteoblast differentiation.**  Cells were cultured as described in the materials and methods. Pegylated HBD1 (Peg HBD1) (1mg/ml) was added when fresh differentiation medium was applied. For PTH treatment, cells were exposed to rat PTH (rPTH, Bachem, 50ng/ml) or bovine PTH (bPTH, Millpore Sigma, 50 ng/ml) for 6 hr then PTH was removed by applying the fresh differentiation medium every 72hr. After 5 cycles of treatment, cell lysates were harvested and immunoblotted with an anti-osteocalcin antibody. The blots were reprobed with an anti-β-actin antibody as a loading control. The bar graph shows the fold increases over the control (Ctrl) based on the ratios of scanning densitometry values of osteocalcin (OC) divided by the β-actin. P<0.05 indicates the significant differences. P, NS indicates no significant differences.
